# SARS-CoV-2 testing strategy: A comparison of restricted and extended strategies in a Swiss outpatient cohort from the community and hospital employees

**DOI:** 10.1371/journal.pone.0250021

**Published:** 2021-04-22

**Authors:** Stéphanie Baggio, Hervé Spechbach, Nathalie Vernaz, Idris Guessous, Laurent Gétaz, Laurent Kaiser, François Chappuis, Julien Salamun, Frédérique Jacquerioz

**Affiliations:** 1 Division of Prison Health, Geneva University Hospitals, Geneva, Switzerland; 2 Office of Corrections, Department of Justice and Home Affairs of the Canton of Zurich, Zurich, Switzerland; 3 Division of Primary Care, Geneva University Hospitals, Geneva, Switzerland; 4 Medical Direction, Geneva University Hospitals, Geneva, Switzerland; 5 Division of Tropical and Humanitarian Medicine, Geneva University Hospitals, Geneva, Switzerland; 6 Geneva Centre for Emerging Viral Diseases, Geneva University Hospitals, Geneva, Switzerland; 7 Division of Infectious Diseases, Geneva University Hospitals, Geneva, Switzerland; 8 Laboratory of Virology, Geneva University Hospitals, Geneva, Switzerland; 9 Division of Laboratory Medicine, Geneva University Hospitals, Geneva, Switzerland; University of Hong Kong, HONG KONG

## Abstract

**Background:**

Testing is a key measure to control the spread of severe acute respiratory syndrome coronavirus 2 (SARS-CoV-2). Here, we empirically compared two SARS-CoV-2 testing strategies.

**Methods:**

We used data from a Swiss single-centre, outpatient cohort study (n = 6,331 test results). A “restricted” strategy was applied to individuals with respiratory symptoms and/or fever and selected risk factors, or an epidemiological link and an “extended” strategy included any clinical symptoms without restriction, irrespective of risk factors and exposure. Data on infection, symptoms, viral load were collected during the first wave (March 11-April 21, 2020) and patients were followed up for clinical complications and hospitalisations until August 31, 2020.

**Findings:**

Infection, clinical complications, and hospitalisation rates were lower for those in the extended strategy compared with the restricted strategy (17.2% *vs*. 25.0%, 12.3% *vs*. 20.8%, and 0.7% *vs*. 2.3%). In the whole cohort, participants included in the extended strategy had a lower number of symptoms (3.51 *vs*. 4.57; p < .001) and visits occurred earlier after symptom onset (0–3 days: 59.2% *vs*. 44.2%; p < .001). Among positive cases, the viral load was higher for the extended strategy (p < .001).

**Conclusions:**

These findings highlighted the crucial importance to implement a widespread testing strategy to achieve a better understanding of the infection, to mount an effective control response, by capturing people when their viral load is highest. A widespread test strategy should be available without barriers to help break the chains of transmission.

## Introduction

The severe acute respiratory syndrome coronavirus 2 (SARS-CoV-2), identified in January, 2020, caused a worldwide outbreak and was declared a pandemic by WHO on March 11, 2020 [1]. By September 30, 2020, over thirty one million cases have been reported worldwide and more than one million deaths [2]. Testing is a key measure to control the epidemic, to reduce the spread of infection [3], to prepare and adapt the public health response [4], and can be used as an exit strategy [5–8]. While some countries have deployed early widespread SARS-CoV-2 testing [9], this strategy has been delayed in other countries with dramatic consequences [9, 10].

At the beginning of the first wave of the epidemic, no widespread testing strategy was used. As clinicians and researchers were unaware of the wide clinical spectrum of symptoms related to COVID-19, testing strategies were generally oriented toward more serious conditions [11, 12]. Later, when the number of cases increased exponentially, more serious conditions remained a target due to testing capacity issues [9] and overwhelmed public health response [13]. To date, there is no empirical support for the potential benefits of a widespread testing strategy. Some modeling studies have shown that population-wide testing reduced the spread of the epidemic [5, 14–17].

The aim of our study was to compare two testing strategies used in Switzerland, a country with one of the highest numbers of SARS-CoV-2 infection per capita worldwide [6]: a “restricted” strategy, based on a specific symptomatology, risk factors, and exposure; and an “extended” strategy, based on any kind of symptoms, irrespective of risk factors or exposure to the disease. We compared rates of infection, clinical complications, and hospitalisations, as well as symptomatology of individuals in each strategy.

## Materials and methods

### Study design and setting

We did a single-centre cohort study of outpatients presenting for SARS-CoV-2 testing at the primary case medicine department of Geneva University Hospitals (HUG), Geneva, Switzerland. Data were collected during the first wave of the epidemic, between March 11, 2020 and April 21, 2020. Information on clinical complications and hospitalisation were collected until August 31, 2020.

HUG is one of the largest university hospitals in Switzerland and in Europe with 2,008 beds and 11,945 healthcare workers. It serves a population of 500,000 residents. In 2019, 64,000 hospitalisations, one million outpatient visits, and 125,000 emergency visits were recorded. HUG was the sole hospital in the canton of Geneva admitting COVID-19 patients. On March 2, 2020, four outpatient SARS-CoV-2 dedicated testing centres were opened by the HUG and patients were oriented to these centres, or to the emergency department for the more severe cases. Individuals tested in private clinics, at the emergency department, and in nursing homes were not included in the study. A standardised questionnaire was completed by a nurse, a trained medical student, or a doctor. As knowledge on the infection evolved over time, the questionnaire was changed accordingly. Differences are explained in the Measures subsection. All patients completed the same questionnaire. The nurse/trained medical student/doctor was not blinded to the patient’s group (restricted and extended strategies), because they need to decide whether the patient should be tested or not.

The Cantonal Ethics Research Committee of Geneva approved the study protocol (no. 2020–00813). Following the article 34 of the Swiss Human Research Act, we were allowed to use health-related personal data collected in routine medical files in the absence of informed consent. Patients who refused the use of their medical data for research purposes were excluded (as documented in the medical file or stated to the medical team during the visit).

### Participants

The recruitment was solely based on patients attending to testing centres during the study period. Patients with severe symptoms were oriented to the emergency department rather than the outpatient testing centres. Exclusion criteria were: 1) being less than 16 years old, 2) refusal of the use of their medical data for research purposes, and 3) exceptions to the testing strategies described below. We also excluded hospitalised infected cases who were non-resident in the canton of Geneva from the analyses on clinical complications, as they could have been followed-up or hospitalised elsewhere.

Participants included the general population (restricted strategy) vs. individuals working at HUG (all employees with and without contact with patients) and healthcare workers in the canton of Geneva (extended strategy, see below).

### Testing strategies

#### Restricted strategy

The testing strategy for the general population was conducted in line with the recommendations of the Swiss national health authorities [18]. Individuals with cough and/or fever were tested.

#### Extended strategy

A more liberal strategy was used for all individuals working at HUG and healthcare workers. They were offered to be tested for any clinical symptoms without restriction. All individuals working at HUG or healthcare workers of the canton of Geneva were classified in the extended strategy, even those meeting the criteria for the restricted strategy (i.e., cough and/or fever).

In both strategies, during a short period (March 18, 2020, to March 22, 2020), access to testing was reduced to individuals with cough, fever, and risk factors due to kit/reagent shortage.

### Measurements

SARS-CoV-2 infection was detected by RT-PCR performed on naso- or oropharyngeal swabs and processed in the HUG virology laboratory, the Swiss national reference laboratory for SARS-CoV-2. Swabs were placed in a collection tube with 150 μL of virus preservation solution. Viral RNA was measured by the cycle threshold (Ct) values targeting the E gene on different diagnostic platforms (Roche COBAS 6800, BD max, Roche Diagnostics, Rotkreuz, Switzerland). Results of the RT-PCR were given back as positive or undetected. Ct values were available for n = 500 positive cases, those tested with Roche COBAS 6800. Ct values were not systematically registered with other diagnostic platforms, especially at the beginning of the pandemic. Ct values are inversely proportional to viral load. Patients with undetected RT-PCR, but strong clinical evidence for SARS-CoV-2 infection, were classified as “probable” cases and counted as positive cases for this study. Finally, some participants were not tested as they did not meet the recommendations for the testing strategy (e.g., no symptom or general population with symptoms but not filling the criteria for being tested).

The triage questionnaire included several symptoms compatible with COVID-19 (not necessarily used to decide whether the patient should undergo a test): anosmia, runny nose, sore throat, cough, difficulty breathing, fever, muscle pain, chills, headache, abdominal symptoms, fatigue, and thoracic pain. Anosmia and headache were added on March 24, 2020. Difficulty breathing, abdominal symptoms, fatigue, and thoracic pain were added on April 1, 2020. These symptoms were reported as free text in the section “other symptoms” in previous versions of the questionnaire and then recoded. We also collected the date of onset of symptoms, which enabled us to identify the time between first symptoms and visit to the testing centres (coded as early visits [0–3 days], or not [4 days or more], and missing values).

Risk factors for severe illness or complications were defined accordingly to Swiss and international recommendations and included: age 65 years or older; hypertension; chronic respiratory disease (e.g., asthma, chronic obstructive pulmonary disease), diabetes, heart diseases (e.g., coronary heart disease, stroke), and immunosuppression (e.g., organ transplant, cancer, immunosuppressive treatment). Participants were asked whether they had a contact with a confirmed SARS-CoV-2 case or travelled abroad in the previous 14 days. Age, gender, and country/canton of residence were also assessed.

COVID-19-related hospitalisation at HUG was collected from patients’ medical records. Patients were hospitalised if they had a high severity score for pneumonia (CURB-65 score ≥ 2), a need or increased need of oxygen, sustained tachypnoea, a poorly-compensated comorbidity, deteriorated general condition, or worsening disease. Criteria for hospitalisation remained the same during the entire study period. According to the local guidelines, mild and moderate pneumonia, not filling the aforementioned criteria, were treated and followed as outpatients and not hospitalised.

COVID-19-related clinical complications were assessed using hospitalisations (see above) and any COVID-19-related visit to the hospital. These visits included worsening, recurrence or persistence of COVID-19-related symptoms, as well as visits planned to monitor severe COVID-19 symptoms (e.g., pneumonia).

### Statistical analysis

Data were analysed at the visit level. We first computed descriptive statistics for all variables. We also performed preliminary analyses comparing demographics, risk factors, number of visits, and exposure between groups, using Chi-square tests with Cramer’s V, linear regressions with R^2^, and negative binomial regressions with pseudo R^2^. We then investigated whether there were differences between the two strategies in the proportion of infected cases, using Chi-square tests with Cramer’s V. We reported likelihood Chi-square contributions and 95% confidence intervals (CI) for the prevalence rates. The relationship between strategies and symptoms was tested using logistic (no symptoms, time to visit after symptom onset) and negative binomial (number of symptoms) regressions. We also performed the same analyses on the subsample of participants who tested positive. Finally, we tested the relationship between strategies and Ct values using linear regression.

We provided a description of the hospitalisation and clinical complications rates with 95% CI for each strategy and tested the difference using a Chi-square test with Cramer’s V. The same analyses were performed on the subsample of participants who tested positive or who were probable cases.

We ran several sensitivity analyses. First, we analysed data at the level of the visit as some patients had repeated visits. We ran all the analyses described above (except for hospitalisation and clinical complications: no clustered observations) taking into account the clustering using mixed-effects regression models. As the results were similar, analyses at the level of the visit are presented. Second, as the standardised questionnaire was not the same during the whole study period, we ran sensitivity analyses on the period for which all symptoms were listed in the questionnaire (starting April 1, 2020). Results were also similar. In the analyses of clinical complications, we also performed sensitivity analyses 1) using the continuous age instead of including age ≥ 65 years in the number of risk factors and 2) excluding asymptomatic patients. Results were again similar. Then, we conducted analyses without probable cases to ensure that the results were the same. Finally, we replicated all analyses on a second subset of participants to control whether there was an effect of being a health care worker. We restricted the extended strategy to the subsample of participants not in contact with patients (n = 250 for the n = 1,606 participants for “contact with patients” was included as a question in the triage questionnaire). Results were similar as those reported in the Results section, except for Ct values for which we could expect a lack of power (n = 18 in the extended strategy). All analyses were done with Stata version 15.

## Results

We registered 7,401 visits to the dedicated testing centres. Forty-two participants (47 visits; 0.6%), were excluded because they did not consent to the use of their data for research purposes. Of the 7,354 remaining visits, 1,023 (13.9%) were excluded because they were exceptions to the testing strategies (n = 792 for the restricted strategy and n = 213 for the extended strategy). A total of 6,331 visits were included in our analysis (restricted testing strategy, 3,023; extended strategy, 3,308), with a mean age of 41.2 ± 12.9 years and 62.6% of females. These corresponded to 2,956 participants for the restricted strategy and 2,998 for the extended strategy. Participants in the extended strategy were more likely to come several times: 9.4% came at least two times versus 2.2% of those in the restricted strategy (p < .001; Table 1). Details are provided in Table 1.

**Table 1:** Sample characteristics (n = 6,331).

		Restricted strategy	Extended strategy	p-value	Effect size
		n = 3,023	n = 3,308		
No. of participants (%, n)^a^					
	1 visit	97.8 (2,956)	90.6 (2,998)	< .001	0.151
	2 or more visits	2.2 (67)	9.4 (310)		
Age (mean, standard deviation)^b^		42.21 (14.39)	40.43 (11.23)	< .001	0.005
Gender (%, n)^a^					
	Female	54.2 (1,639)	70.3 (2,324)	< .001	0.166
	Male	45.8 (1,384)	29.7 (984)		
Residence (%, n)^a^					
	Geneva	89.5 (2,706)	60.2 (1,989)	< .001	0.338
	Another canton in Switzerland	2.1 (62)	4.2 (139)		
	France	8.4 (254)	35.6 (1,179)		
Risk factors (%, n)^a^					
	Age ≥ 65	7.2 (219)	0.0 (1)	-	-
	Hypertension	10.9 (329)	5.4 (179)	< .001	0.101
	Chronic respiratory disease	15.1 (455)	11.9 (392)	< .001	0.047
	Heart disease	4.1 (124)	1.8 (59)	< .001	0.069
	Immunosuppression	5.8 (174)	2.7 (89)	< .001	0.077
	Diabetes	4.8 (145)	1.8 (59)	< .001	0.085
	Total no. of risk factors (mean, standard deviation)^c^	0.48 (0.82)	0.24 (0.50)	< .001	0.023
Exposure^a^					
	Travelled abroad	8.9 (268)	10.0 (330)	.131	-
	Contact with a confirmed SARS-CoV-2 case	29.0 (876)	59.3 (1,962)	< .001	0.305

^a^ χ^2^ and Cramer’s V.

^b^ Linear regression and R^2^.

^c^ Negative binomial regression and pseudo R^2^.

For the restricted strategy, 25.0% (n = 756; 95% CI: 23.5–26.6) of visits were infected, including 3 probable cases. A total of 8.2% (n = 249) of visits did not lead to a RT-PCR. For the extended strategy, 17.2% (n = 568; 95% CI: 15.3–17.8) of visits were infected (including 5 probable cases). A total of 4.1% (n = 136) of visits did not lead to a RT-PCR. There was a significant difference between strategies *(χ*^*2*^: 121.57; p < .001; Cramer’s V: 0.139), with the extended strategy resulting in a lower number of visits with a positive RT-PCR (likelihood χ^2^ ratio contribution: -224.0), a higher number of visits with a negative RT-PCR (likelihood χ^2^ ratio contribution: 392.3), and no RT-PCR (likelihood χ^2^ ratio contribution: -106.5).

Table 2 provides detailed information on associations of strategies with symptoms and Ct values. The total number of symptoms was significantly lower for the extended strategy (3.51 *vs*. 4.57 symptoms on average; p < .001). Participants from the extended strategy were more likely to come in the three first days after symptoms onset (59.2% *vs*. 44.2%, p < .001). Among positive cases for which Ct values were available (n = 500), Ct values were significantly lower in the extended strategy in comparison with the restricted strategy (24.49 *vs*. 27.62, p < .001).

**Table 2:** Associations between testing strategies and symptoms (n = 6,331).

		Restricted strategy	Extended strategy	Estimate (95% confidence intervals)	p-value
Total no. of symptoms^a^		4.57	3.51	-0.26 (-0.29; -0.24)	< .001
Time to visit after symptom onset^b^					
	Early visit: 0–3 days (ref.)	44.2	59.2	-	-
	4 days or more	51.5	32.3	-0.76 (-0.87; -0.66)	< .001
	Missing values	4.2	8.5	0.41 (0.19; 0.63)	< .001
Ct values^c^		27.62	24.49	-3.13 (-4.27; -2.00)	< .001

The restricted strategy is used as the reference category.

^a^ Negative binomial regression (means and b estimate are reported).

^b^ Multinomial logistic regression (percentages and b estimates are reported). Missing values: n = 410 (6.5%).

^c^ Linear regression (means and b estimates are reported). The analysis was performed on n = 500 positive cases for which Ct values were available.

The overall hospitalisation rate of patients resident in Geneva (n = 4,695) was 2.3% (n = 62; 95% CI: 1.8–2.9) for the restricted strategy and 0.7% (n = 14; 95% CI: 0.4–1.2) for the extended strategy. Among positive or probable cases (n = 1,022) of Geneva residents, 9.1% (95% CI: 7.2–11.6) of the participants were hospitalized for the restricted strategy and 4.1% (95% CI: 2.4–6.8) for the extended strategy; 20.8% (n = 141, 95% CI: 17.9–24.0) had clinical complications for the restricted strategy and 12.3% (n = 42, 95% CI: 9.2–16.2) for the extended strategy.

## Discussion

This study compared two testing strategies to provide empirical support to the potential benefits of widespread SARS-CoV-2 testing. Our findings showed that the infection rate (17.2% *vs*. 25.0%) and hospitalisation rate (whole sample: 0.7% *vs*. 2.3%; SARS-CoV-2 positive subsample: 4.1% *vs*. 9.1%) were lower for the extended strategy compared with the restricted strategy. More restricted testing strategies are skewed towards more severe cases requiring hospitalisation and, therefore, do not provide a good overview of the detrimental consequences of the disease at the population level [12]. From a public health point of view, our results empirically support that extended testing is required to provide an accurate picture of the disease spectrum and severity.

In the extended strategy, visits were more likely to occur during the first days after symptom onset. As the viral load is higher at the beginning of the disease [19], early testing may help to reduce the spread of infection. The significantly lower Ct values for the extended strategy in comparison with the restricted strategy suggested that viral loads were higher for patients benefiting of the extended strategy. In addition, in the extended strategy visits with milder symptoms or no symptoms were more frequent compared with the restricted strategy. Identifying these pauci-symptomatic cases is crucial to control the spread of infection and help break the chains of transmission by providing adequate recommendations for self-isolation for infected cases and quarantine for their contacts [5]. When applied to healthcare workers, it also protects patients and decreases absenteeism [20]. These findings are in line with previous mathematical models reporting a reduction in transmission by 25–33% with a widespread testing strategy [15], or even 40% [17]. As this finding was not replicated in our sensitivity analysis, probably because a lack of power, these results need to be confirmed in further empirical studies.

Of note, an extended testing strategy can also have potential negative sides. For example, people are more likely to come several times, resulting in increased healthcare costs and overcrowding of testing centres. In addition, individuals may follow preventive measures (social distancing, stay-at-home messages) less carefully because they can access testing easily. To date, these aspects have not been investigated in empirical public health research.

Given the benefits of the widespread strategy from a public health and infection control perspective, barriers to testing should be avoided. In Switzerland, the cost of visit and testing was 310.30 Swiss francs (US$ 334/287 euros) at the beginning of the epidemic and has now become free of charge end of June 2020. Even if Switzerland has universal health care coverage, the cost was not necessarily covered by health insurances because of deductibles and out-of-pocket payments [21, 22]. In countries without universal health coverage, such a situation can lead to inequality in healthcare access and, consequently, epidemic resurgence. Free testing should be also extended to vulnerable populations, such as undocumented migrants or people in detention. These populations live in overcrowded conditions and often lack access to health care, conditions that are likely to favor an outbreak and promote severe detrimental health outcomes due to previous poor health and the presence of undiagnosed chronic diseases.

This study has some limitations. First, information collected in the standardised questionnaire was based on self-reports. Even if questionnaires were always checked by a health care professional, they may have lacked precision. Second, we used different versions of the standardised questionnaire. New symptoms were added when they appeared as frequent SARS-CoV-2 symptoms. Despite the recoding of symptoms listed in the “other symptoms” section, these symptoms might have been underreported in earlier versions of the questionnaire. However, our sensitivity analyses yielded similar results and the standardised questionnaire was the same for the two testing strategies. Finally, the populations for the restricted and extended strategies were not the same. Individuals working at HUG and healthcare workers were more likely to have a good health literacy (“healthy bias”) and testing was more easily available for them (“proximity bias”). This may have inflated the differences between the two testing strategies and studies comparing strategies in the same population are required to achieve a better understanding of their benefits and limits.

## Conclusion

Our results highlight that widespread testing is crucial to understand and control the spread of infection, and to maximize identification of infected people. Access to free testing is essential, not only to achieve infection control, but also to eliminate any discrimination between the different layers of society.

## Supporting information

S1 File (XLSX)
